# Susceptible genes of type 2 diabetes and their disease predictive power in Chinese population

**DOI:** 10.1186/1479-5876-10-S2-A16

**Published:** 2012-10-17

**Authors:** Weiping Jia

**Affiliations:** 1 Shanghai Diabetes Institute, Department of Endocrinology and Metabolism, Shanghai Clinical Center for Diabetes, Shanghai Key Laboratory for Diabetes, Shanghai Jiao Tong University Affiliated Sixth People’s Hospital, Shanghai, China

## 

Type 2 diabetes mellitus is influenced by the environmental factors and genetic factors. Due to the heterogeneity in environmental factors as well as genetic background, the susceptibility of type 2 diabetes mellitus in different ethnic populations is totally different, with low susceptibility in Caucasians, while modest susceptibility in Chinese Hans. The recent success of genetics have identified 50 susceptible genes of type 2 diabetes in the Caucasians by biology candidate study, linkage study as well as genome-wide association study, among which some loci such as *KCNJ11*, *TCF7L2*, *TCF2* genes have been replicated successfully in our Chinese Hans populations with the odds ratio (OR) ranging from 1.14-1.31. Besides, by replicating the susceptible genes specific for the East Asian, 6 loci showed significance in our Chinese samples, with *KCNQ1* showing the strongest association with type 2 diabetes. Furthermore, we have been searching for new susceptibility genes in Han Chinese. We also performed a positional cloning study and found that one of the nominal associations was located in the gene NOS1AP. We also used GWAS to identify susceptible genes of the Chinese and identified eight novel hits by collaboration with East Asian groups. However, although nearly 50 susceptible genes for type 2 diabetes were identified so far, the prediction effects were still limited. Thus the utility of both genetic and environmental factors will be worthy to explore in the disease prediction.

